# Information Model for Machine-Tool-Performance Tests

**DOI:** 10.6028/jres.106.018

**Published:** 2001-04-01

**Authors:** Y. Tina Lee, Johannes A. Soons, M. Alkan Donmez

**Affiliations:** National Institute of Standards and Technology, Gaithersburg, MD 20899-0001

**Keywords:** data exchange, EXPRESS language, information model, machine performance test, machine tools, standard for the exchange of product model data

## Abstract

This report specifies an information model of machine-tool-performance tests in the EXPRESS [1] language. The information model provides a mechanism for describing the properties and results of machine-tool-performance tests. The objective of the information model is a standardized, computer-interpretable representation that allows for efficient archiving and exchange of performance test data throughout the life cycle of the machine. The report also demonstrates the implementation of the information model using three different implementation methods.

## 1. Introduction

### 1.1 Objective

This report specifies an information model of machine-tool-performance tests in the EXPRESS modeling language [1]. It is based on the information model described in the Data Specification for Machine Tool Performance Tests, Version 2.3e [2]. The objective of the information model is a standardized, computer-interpretable representation that allows for efficient archiving and exchange of performance test data throughout the life cycle of a machine tool. It serves as a basis for generating database schemas, database calls, and neutral file formats. Performance test data of machine tools is used for machine acceptance, performance tracking, software compensation, and to evaluate the capability of a machine to manufacture a part to specified tolerances.

The information model specifies the test procedure, the test conditions, the equipment, the measurement set-up, and the test results. The model can be used to describe the properties and results of a performance test at a level close to the raw measurement data. The information elements also enable the user to re-create the set-up, equipment settings, and measurement procedure. The model captures key information on the large variety of possible test set-ups and measurement procedures, which is essential for the interpretation of the test results. A subset of the specification can be used to summarize the test, focusing on performance parameters that are estimated from the measurement results.

The information model addresses machine tool properties that are verified by performance tests. It complements machine-tool-specification data that is not tested, e.g., the machine configuration, the workspace, weight and size of the machine, tool holder standard, auxiliary devices, etc. [3].

The information model is intended to serve as the starting point for a future, standardized representation. The model is expected to change and grow based on further review and future implementation experience.

This report is structured as follows: In the remainder of Sec. 1 the problem statement and scope of the information model are defined; modeling language and implementation methods are identified. In Sec. 2 the information model is presented. In Sec. 3 three sample data files are provided. In Sec. 4 software tools that support the implementation of EXPRESS information models are briefed. Section 5 presents the conclusion of this report. An appendix is provided for describing a set of EXPRESS keywords in Sec. 6. The final section lists the references used in this report.

### 1.2 Problem Statement

Today’s manufacturing industry greatly relies on computer technology to support activities throughout a product’s life cycle. Efficient and distributed access to the performance data of machine tools is important in manufacturing. The results of performance tests are used for machine acceptance, predictive maintenance, error compensation, and to evaluate the capability of a machine to manufacture parts to specified tolerances. A critical enabler to the efficient interchange and storage of performance data is a unified information model for the results and properties of performance tests.

Currently, there is no agreed-upon mechanism for representing the properties and results of machine-tool-performance tests [2]. There exists a variety of software packages for the performance evaluation of machine tools. They usually have been developed by the manufacturer of a particular measurement device, such as a laser interferometer or a ball bar, and are tailored to that particular instrument. The software packages employ different data models and store the data in files using vendor-specific formats. This complicates data exchange, data storage in databases, and use of the data by third-party software. Furthermore, the stored data is often limited to the data required to produce the graphs and numbers specified in the various standards for machine-tool-performance evaluation (e.g., [4,5,6]). This may result in inefficient access or even loss of the additional data that is required for other applications, such as virtual machining and software error compensation. Finally, not all tests described in the standards are addressed by existing software for machine tool testing. This is often the case for tests that require generic equipment, such as displacement indicators. Users have created their own “in-house” methods, often using spreadsheets, to store the properties and results of these tests, often on an ad-hoc basis.

### 1.3 Scope

This specification supports the majority of instrumented, machine-tool-performance tests defined in the American [4,5] and international [6] standards:

1. Positioning accuracy and repeatability of linear and angular positioning axes.
2. Geometric errors of linear and angular positioning axes.
3. Spindle axis of rotation.
4. Machine thermal tests: environmental temperature variation error (ETVE), spindle, axis, and composite.
5. Critical alignments: parallelism and squareness of machine axes.
6. Circular contouring tests.
7. Diagonal displacement tests.
8. Subsystem repeatability (tool change, turret, gage line, and pallet repeatability).
9. Compliance and hysteresis.

For these tests the following information is described:

1. Date and time of the test.
2. Identification of the machine tool on which the test was performed.
3. Indication as to why the test was performed.
4. The operator who performed the test.
5. The machine status and environmental conditions during the test.
6. The standard in which the test is defined.
7. The equipment and software used to perform the test.
8. The measurement set-up and operating parameters.
9. The raw measurement data.
10. The calculated performance parameters.

### 1.4 Modeling Language and Implementation Methods

The information model presented in this report is in the EXPRESS language. The EXPRESS modeling language [1] was developed as part of the International Organization for Standardization (ISO) Standard 10303, commonly known as the Standard for the Exchange of Product Model Data (STEP) [7]. STEP is the result of an effort to develop a mechanism for digitally representing the physical and functional characteristics of a product throughout the product’s life cycle. STEP includes information models and mechanisms for representing the models and related data. EXPRESS is a formally specified structured language. EXPRESS models have an object-oriented flavor. The reason EXPRESS is chosen here is three-fold: EXPRESS is primarily an information modeling language, EXPRESS is a textual representation that permits machine processing of the specification, and EXPRESS consists of language elements that allow unambiguous object definitions and specification of constraints on the objects defined.

An information model provides a sharable, stable, and organized structure of information requirements. It is developed to be independent from implementations using different data format structures. Implementation independence allows users to select their implementation methods. The selection of an implementation method is heavily dependent on the target environment where the application system resides, but the information contained in the model is not dependent on the target environment if the modeling is done properly. Currently, the implementation methods used by the manufacturing community include:

1. data transfer via a working form, which is a structured, in-memory representation of data.
2. data transfer via an exchange file, which is a file with a predefined structure or format.
3. data transfer using a database management system [8].

STEP introduced the 10303 Exchange Structure in ISO 10303-21, or the *Part 21* file, as an implementation method for actual EXPRESS models [9]. A *Part 21* file contains instances of the various entities defined by the EXPRESS information model. The *Part 21* file format is just one of the implementation methods for representing data in accordance with the EXPRESS information models. Tools that support the implementation of EXPRESS information models are briefly described in Sec. 4.

## 2. Information Model

In this section, an EXPRESS information model for representing the properties and results of machine-tool-performance tests is presented. Section 2.1 describes the structure of data requirements. The schema is presented in detail in Sec. 2.2. Appendix A contains the listing of EXPRESS keywords that are used in the schema.

### 2.1 Structure of Data Requirements

The information model presented in Sec. 2.2 is based on the “Data Specification for Machine Tool Performance Tests, Version 2.3e” [2]. The large variety of addressed performance tests are classified into four groups:

**Table:** 

1) Circular:	tests where error motions are measured at points on a circular path in the machine workspace.
2) Line:	tests where error motions are measured at points on a line in the space spanned by the positioning axes of the machine (e.g., positioning accuracy, axis geometry, diagonal displacement accuracy, axis alignment, and thermal distortion caused by axis motion).
3) Point:	tests where error motions are measured at a single point in the space spanned by the positioning axes of the machine (e.g., subsystem repeatability, spindle axis of rotation, spindle thermal stability, and Environmental Temperature Variation Error).
4) Compliance:	tests for the compliance and hysteresis of the machine under static loads.

**Fig. 1:**
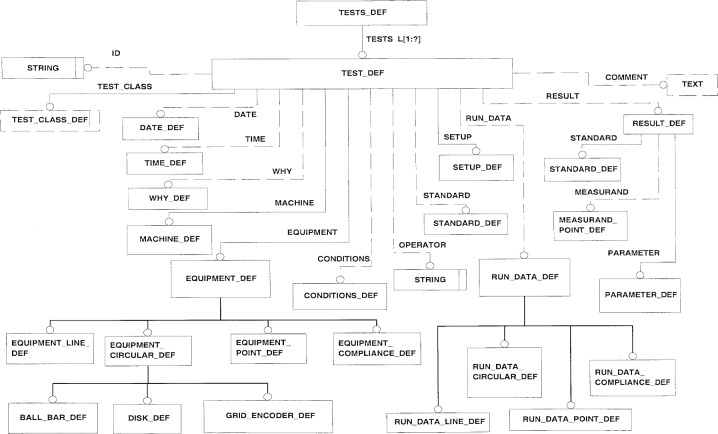
Overview of the Entities relationships.

The specifications for other performance tests, e.g., computer numerical control (CNC) performance tests, machining tests, and tests addressing the measurement capabilities of a machine tool, are under development and will follow the structure outlined below. Figure 1 shows the relationships among the major entities in the information model. The figure is presented by EXPRESS-G^1^ [1], a graphical subset of the EXPRESS language. The TESTS entity is a list of TEST entities, each describing the properties and results of a performance test. The TEST entity contains the MACHINE entity identifying the tested machine. This is achieved by either a unique identification (ID) or a set of properties: manufacturer, model, and serial number. A unique ID as an alternative to a set of properties is also given to several other entities in the model.

Most of the parameters that describe the design of a test are contained in three entities: CONDITIONS, EQUIPMENT, and SETUP. The CONDITIONS entity describes the status of the machine and its environment during the test. The parameters of this entity apply to most tests. By contrast, the content of the EQUIPMENT and SETUP entities varies depending on the type of test. The EQUIPMENT entity describes the properties and (factory) settings of a kit of instruments and artifacts assembled for a specific type of test. The entity usually does not change once such a kit has been defined. The SETUP entity contains parameters that describe the tested machine property, the set-up, and the measurement procedure. The parameter values usually vary unless a test is repeated. The majority of the information contained in the SETUP entity is not dependent on the content of the EQUIPMENT entity.

The results of a test are contained in two entities: RUN_DATA and RESULT. RUN_DATA contains the measurement data of an individual run. A run is a specific motion pattern of the machine during which errors are measured. A performance test usually consists of several runs that can only differ in the approach direction to the target points. A RESULT entity contains performance parameters that are estimated from the data obtained in one or more runs. A test can have multiple RESULT entities that may differ because of differing physical testing conditions, such as direction of probe approach.

The use of a particular system of measurement units is site-specific. However, use of mixed units will complicate the exchange and storage of data. Therefore, the units of measurement values used in this information model are predefined [2]. It is assumed that the application software will make the desired conversions to and from these units.

### 2.2 EXPRESS Information Model

This subsection describes the detailed information for the schema of machine-tool-performance tests. The schema name is MACHINE_TOOL_PERFORMANCE_TESTS. An EXPRESS schema is composed of declarations of types, entities, constraints, and their relationships. The concept of a type in EXPRESS is the same as that of a data type in a standard programming language. It defines the kind of values that an object may assume. Entities are the focal point of an EXPRESS information model. An entity declaration describes the information content of an object, as well as some of the constraints on the objects.

In EXPRESS language, a “remark” is used for documentation and is not significant as a language element. The character pair, “(” and “*”, is used to denote the start of an embedded remark, and the character pair, “*” and “)”, is used to denote its end. This character pair is also known as a “token.” An embedded remark may appear between any two tokens. In this report, the documentation is presented as embedded remarks. Consequently, if we were to begin the report with “(*”, the entire report could be read into an EXPRESS parser for further analysis.


*)
*SCHEMA MACHINE_TOOL_PERFORMANCE_TESTS;*
(*


#### 2.2.1 Entity Definitions

The entities are formally defined in this subsection. The entities presented here are in the “top-down” order, i.e., primitive type definitions are presented last.


 *)
*ENTITY TESTS_DEF;*
 *TESTS: LIST [1:?] OF UNIQUE TEST_DEF; END_ENTITY;*
*ENTITY TEST_DEF;*
 *ID: OPTIONAL STRING;*
 *TEST_CLASS: TEST_CLASS_DEF;*
 *DATE: DATE_DEF;*
 *TIME: OPTIONAL TIME_DEF;*
 *WHY: OPTIONAL WHY_DEF;*
 *MACHINE: MACHINE_DEF;*
 *CONDITIONS: OPTIONAL CONDITIONS_DEF;*
 *OPERATOR: OPTIONAL STRING;*
 *STANDARD: OPTIONAL STANDARD_DEF;*
 *EQUIPMENT: EQUIPMENT_DEF;*
 *SETUP: SETUP_DEF;*
 *RUN_DATA: OPTIONAL LIST [1:?] OF RUN_DATA_DEF;*
 *RESULT: OPTIONAL LIST [1:?] OF RESULT_DEF;*
 *COMMENT: OPTIONAL TEXT;*
*END_ENTITY;*
*ENTITY DATE_DEF;*
 *YYYY: INTEGER;*
 *MM: INTEGER;*
 *DD: INTEGER;*
  *WHERE*
  *WR1: (YYYY = 1900);*
  *WR2: (1 <= MM) AND (MM <= 12);*
  *WR3: (1 <= DD) AND (DD <= 31);*
*END_ENTITY;*
*ENTITY TIME_DEF;*
 *HH: INTEGER;*
 *MM: INTEGER;*
 *SS: INTEGER;*
  *WHERE*
  *WR1: (0 <= HH) AND (HH <= 24);*
  *WR2: (0 <= MM) AND (MM <= 59);*
  *WR3: (0 <= SS) AND (SS <= 59);*
  *WR4: ((HH = 24) AND ((MM = 0) AND (SS = 0)));*
*END_ENTITY;*
*ENTITY MACHINE_DEF;*
 *ID: OPTIONAL STRING;*
 *MANUFACTURER: STRING;*
 *MACHINE_MODEL: STRING;*
 *SERIAL_NUMBER: STRING;*
 *LOCATION: OPTIONAL STRING;*
*END_ENTITY;*
*ENTITY CONDITIONS_DEF;*
 *CLAMPED_AXES: OPTIONAL LIST [1:?] OF AXIS_DEF;*
 *COMPENSATION: OPTIONAL BOOLEAN;*
 *COMPENSATION_ID: OPTIONAL STRING;*
 *COOLANT: OPTIONAL BOOLEAN;*
 *DRIVE_STATUS: OPTIONAL DRIVE_STATUS_DEF;*
 *TEMP_ENVIRONMENT: OPTIONAL REAL;*
 *WARMUP: OPTIONAL BOOLEAN;*
 *WARMUP_DESCRIPTION: OPTIONAL STRING;*
*END_ENTITY;*
*ENTITY STANDARD_DEF;*
 *ORGANIZATION: STRING;*
 *STANDARD_NUMBER: STRING;*
 *NAME: OPTIONAL STRING;*
 *YEAR: INTEGER;*
 *TEST_NAME: OPTIONAL STRING;*
 *SECTION_NUMBER: OPTIONAL STRING;*
 *SECTION_NAME: OPTIONAL STRING;*
 *STATUS: OPTIONAL STANDARD_STATUS_DEF;*
  *WHERE*
  *WR1: (YEAR>1900);*
*END_ENTITY;*
*ENTITY EQUIPMENT_DEF*
 *SUPERTYPE OF (ONEOF (EQUIPMENT_CIRCULAR_DEF*,
   *EQUIPMENT_LINE_DEF*,
   *EQUIPMENT_POINT_DEF*,
   *EQUIPMENT_COMPLIANCE_DEF));*
  *ID: OPTIONAL STRING;*
  *COMPONENT: OPTIONAL LIST [1:?] OF COMPONENT_DEF;*
  *SOFTWARE: OPTIONAL SOFTWARE_DEF;*
  *RESOLUTION: OPTIONAL REAL;*
  *SAMPLE_RATE_RAW: OPTIONAL REAL;*
*END_ENTITY;*
*ENTITY COMPONENT_DEF;*
 *ID: OPTIONAL STRING;*
 *DESCRIPTION: OPTIONAL STRING;*
 *MANUFACTURER: STRING;*
 *COMPONENT_MODEL: STRING;*
 *SERIAL_NUMBER: STRING;*
 *CALIBRATION_DATE: OPTIONAL DATE_DEF;*
 *CALIBRATION_EXP_DATE: OPTIONAL DATE_DEF;*
 *CERTIFICATE_NUMBER: OPTIONAL STRING;*
 *CALIBRATION_ORGANIZATION: OPTIONAL STRING;*
*END_ENTITY;*
*ENTITY SOFTWARE_DEF;*
 *ID: OPTIONAL STRING;*
 *MANUFACTURER: STRING;*
 *NAME: STRING;*
 *VERSION_NUMBER: STRING;*
*END_ENTITY;*
*ENTITY SETUP_DEF*
 *SUPERTYPE OF (ONEOF (SETUP_CIRCULAR_DEF*,
   *SETUP_LINE_DEF*,
   *SETUP_POINT_DEF*,
   *SETUP_COMPLIANCE_DEF));*
*END_ENTITY;*
*ENTITY RUN_DATA_DEF*
 *SUPERTYPE OF (ONEOF (RUN_DATA_CIRCULAR_DEF*,
   *RUN_DATA_LINE_DEF*,
   *RUN_DATA_POINT_DEF*,
   *RUN_DATA_COMPLIANCE_DEF));*
*END_ENTITY;*
*ENTITY RESULT_DEF;*
 *STANDARD: STANDARD_DEF;*
 *MEASURAND: OPTIONAL MEASURAND_POINT_DEF;*
 *PARAMETER: LIST [1:?] OF PARAMETER_DEF;*
*END_ENTITY;*
*ENTITY PARAMETER_DEF;*
 *NAME: STRING;*
 *VAL: REAL;*
 *APPROACH_DIRECTION: OPTIONAL APPROACH_DIRECTION_DEF;*
*END_ENTITY;*
*ENTITY EQUIPMENT_CIRCULAR_DEF*
 *SUPERTYPE OF (ONEOF(BALL_BAR_DEF*,
  *DISK_DEF*,
  *GRID_ENCODER_DEF))*
 *SUBTYPE OF (EQUIPMENT_DEF);*
  *EQUIPMENT_CLASS: EQUIPMENT_CLASS_CIRCULAR_DEF;*
  *ABSOLUTE: BOOLEAN;*
  *FILTER_LS_CENTER: BOOLEAN;*
  *FILTER_LS_RADIUS: BOOLEAN;*
  *TEMP_REFERENCE_COMP: BOOLEAN;*
  *TEMP_REFERENCE_SENSOR: OPTIONAL LIST [1:?] OF TEMP_SENSOR_DEF;*
  *TEMP_REFERENCE_COEFFICIENT: REAL;*
*END_ENTITY;*
*ENTITY BALL_BAR_DEF*
 *SUBTYPE OF (EQUIPMENT_CIRCULAR_DEF);*
  *CALIBRATOR: OPTIONAL BOOLEAN;*
*END_ENTITY;*
*ENTITY DISK_DEF*
 *SUBTYPE OF (EQUIPMENT_CIRCULAR_DEF);*
  *MACHINE_PROBE: BOOLEAN;*
  *INNER_CIRCLE: OPTIONAL BOOLEAN;*
*END_ENTITY;*
*ENTITY GRID_ENCODER_DEF*
 *SUBTYPE OF (EQUIPMENT_CIRCULAR_DEF);*
*END_ENTITY;*
*ENTITY SETUP_CIRCULAR_DEF*
 *SUPERTYPE OF (ONEOF(SETUP_CIRCULAR_STATIC_DEF*,
  *SETUP_CIRCULAR_DYNAMIC_DEF))*
*SUBTYPE OF (SETUP_DEF);*
  *ID: OPTIONAL STRING;*
  *MEAS_MODE: MEAS_MODE_DEF;*
  *PLANE: PLANE_DEF;*
  *ROTARY_AXIS: OPTIONAL AXIS_DEF;*
  *CENTER: MACHINE_POSITION_DEF;*
  *TOOL_VECTOR: TOOL_VECTOR_DEF;*
  *SPINDLE_NUMBER: OPTIONAL INTEGER;*
  *TURRET_NUMBER: OPTIONAL INTEGER;*
  *RADIUS_MACHINE: REAL;*
  *RADIUS_REFERENCE: REAL;*
  *INCLINATION: REAL;*
  *FEEDRATE: REAL;*
  *OVERSHOOT: OPTIONAL REAL;*
  *TEMP_MATERIAL_COMP: BOOLEAN;*
  *TEMP_MATERIAL_SENSOR: OPTIONAL LIST [1:?] OF TEMP_SENSOR_DEF;*
  *TEMP_MATERIAL_COEFFICIENT: OPTIONAL REAL;*
  *SAMPLES_AVERAGED: OPTIONAL INTEGER;*
  *POINT_MODE: POINT_MODE_CIRCULAR_DEF;*
  *INTERPOLATION: OPTIONAL INTERPOLATION_DEF;*
  *NC_PROGRAM_ID: OPTIONAL STRING;*
  *ALIGNMENT_METHOD: OPTIONAL ALIGNMENT_METHOD_DEF;*
  *ALIGNMENT_WHEN: OPTIONAL ALIGNMENT_WHEN_DEF;*
  *DATUM_WHEN: OPTIONAL DATUM_WHEN_DEF;*
  *PREVIOUS_TEST_ID: OPTIONAL STRING;*
   *WHERE*
   *WR1: (TEMP_MATERIAL_COMP) AND (EXISTS(TEMP_MATERIAL_COEFFICIENT));*
*END_ENTITY;*
*ENTITY PLANE_DEF;*
 *X: AXIS_DEF;*
 *Y: AXIS_DEF;*
*END_ENTITY;*
*ENTITY SETUP_CIRCULAR_STATIC_DEF*
 *SUBTYPE OF (SETUP_CIRCULAR_DEF);*
  *SETUP_STATIC: SETUP_STATIC_DEF;*
  *APPROACH_MODE: APPROACH_MODE_CIRCULAR_DEF;*
*END_ENTITY;*
*ENTITY SETUP_CIRCULAR_DYNAMIC_DEF*
 *SUBTYPE OF (SETUP_CIRCULAR_DEF);*
  *SETUP_DYNAMIC: SETUP_DYNAMIC_DEF;*
  *CAM_SOFTWARE: OPTIONAL SOFTWARE_DEF;*
  *NC_CIRCULARITY: OPTIONAL REAL;*
*END_ENTITY;*
*ENTITY RUN_DATA_CIRCULAR_DEF*
 *SUBTYPE OF (RUN_DATA_DEF);*
  *APPROACH_DIRECTION: APPROACH_DIRECTION_DEF;*
  *LS_CENTER_OFFSET_X: OPTIONAL REAL;*
  *LS_CENTER_OFFSET_Y: OPTIONAL REAL;*
  *LS_RADIUS_ERROR: OPTIONAL REAL;*
  *TEMP_REFERENCE: OPTIONAL LIST [1:?] OF TEMP_DATA_DEF;*
  *TEMP_MATERIAL: OPTIONAL LIST [1:?] OF TEMP_DATA_DEF;*
  *POINTS: LIST[1:?] OF REAL;*
   *WHERE*
   *WR1: ((EQUIPMENT_CIRCULAR_DEF.FILTER_LS_CENTER) AND*
    *(EXISTS (LS_CENTER_OFFSET_X) AND*
    *EXISTS (LS_CENTER_OFFSET_Y)));*
   *WR2: ((EQUIPMENT_CIRCULAR_DEF.FILTER_LS_RADIUS) AND*
    *(EXISTS (LS_RADIUS_ERROR)));*
*END_ENTITY;*
*ENTITY EQUIPMENT_LINE_DEF*
 *SUBTYPE OF (EQUIPMENT_DEF);*
  *EQUIPMENT_CLASS: EQUIPMENT_CLASS_LINE_DEF;*
  *FILTER_LS_SLOPE: OPTIONAL BOOLEAN;*
  *FILTER_LS_CENTER: OPTIONAL BOOLEAN;*
  *FILTER_OFFSET: BOOLEAN;*
  *TEMP_REFERENCE_COMP: OPTIONAL BOOLEAN;*
  *TEMP_REFERENCE_COEFFICIENT: OPTIONAL REAL;*
  *TEMP_REFERENCE_SENSOR: OPTIONAL LIST [1:?] OF TEMP_SENSOR_DEF;*
  *LASER_INTERFEROMETER: OPTIONAL LASER_INTERFEROMETER_DEF;*
  *TARGET_SHAPE: OPTIONAL TARGET_SHAPE_DEF;*
  *TARGET_DIAMETER: OPTIONAL REAL;*
*END_ENTITY;*
*ENTITY LASER_INTERFEROMETER_DEF;*
 *AIR_HUMIDITY_COMP: OPTIONAL BOOLEAN;*
 *AIR_PRESSURE_COMP: OPTIONAL BOOLEAN;*
 *DEADPATH_COMP: OPTIONAL BOOLEAN;*
 *VOL_COMP_METHOD: OPTIONAL VOL_COMP_METHOD_DEF;*
*END_ENTITY;*
*ENTITY SETUP_LINE_DEF*
 *SUPERTYPE OF (ONEOF (SETUP_LINE_STATIC_DEF*,
   *SETUP_LINE_DYNAMIC_DEF))*
 *SUBTYPE OF (SETUP_DEF);*
  *ID: OPTIONAL STRING;*
  *MEAS_MODE: MEAS_MODE_DEF;*
  *MEASURAND: MEASURAND_LINE_DEF;*
  *MEAS_METHOD: MEAS_METHOD_DEF;*
  *AXIS: OPTIONAL AXIS_DEF;*
  *SENSITIVE_DIRECTION: OPTIONAL SENSITIVE_DIRECTION_DEF;*
  *START_POINT: MACHINE_POSITION_DEF;*
  *END_POINT: OPTIONAL MACHINE_POSITION_DEF;*
  *TOOL_VECTOR: TOOL_VECTOR_DEF;*
  *SPINDLE_NUMBER: OPTIONAL INTEGER;*
  *TURRET_NUMBER: OPTIONAL INTEGER;*
  *FEEDRATE: REAL;*
  *DEADPATH: OPTIONAL REAL;*
  *OVERSHOOT: OPTIONAL REAL;*
  *WARMUP_MOVES: OPTIONAL INTEGER;*
  *WARMUP_RUNS: OPTIONAL INTEGER;*
  *TEMP_MATERIAL_COMP: OPTIONAL BOOLEAN;*
  *TEMP_MATERIAL_SENSOR: OPTIONAL LIST [1:?] OF TEMP_SENSOR_DEF;*
  *TEMP_MATERIAL_COEFFICIENT: OPTIONAL REAL;*
  *TEMP_ADDITIONAL_SENSOR: OPTIONAL LIST [1:?] OF TEMP_SENSOR_DEF;*
  *SAMPLES_AVERAGED: OPTIONAL INTEGER;*
  *ALIGNMENT: OPTIONAL ALIGNMENT_DEF;*
  *DIFFERENTIAL_MEAS_DIR : OPTIONAL DIFFERENTIAL_MEAS_DIR_DEF;*
  *SENSOR_OFFSET : OPTIONAL REAL;*
  *REVERSAL: OPTIONAL BOOLEAN;*
   *WHERE*
   *WR1: (((TEST_DEF.TEST_CLASS = TEST_CLASS_DEF.DIAGONAL_ACCELERATION) OR*
    *(TEST_DEF.TEST_CLASS = TEST_CLASS_DEF.DIAGONAL_ANGULAR) OR*
    *(TEST_DEF.TEST_CLASS = TEST_CLASS_DEF.DIAGONAL_POSITION) OR*
    *(TEST_DEF.TEST_CLASS = TEST_CLASS_DEF.DIAGONAL_STRAIGHT) OR*
    *(TEST_DEF.TEST_CLASS = TEST_CLASS_DEF.DIAGONAL_VELOCITY)) AND*
    *(EXISTS(END_POINT)));*
   *WR2: (((TEST_DEF.TEST_CLASS = TEST_CLASS_DEF.DIAGONAL_POSITION) OR*
    *(TEST_DEF.TEST_CLASS = TEST_CLASS_DEF.AXIS_POSITION) OR*
    *(TEST_DEF.TEST_CLASS = TEST_CLASS_DEF.AXIS_REPEAT) OR*
    *(TEST_DEF.TEST_CLASS = TEST_CLASS_DEF.AXIS_REVERSAL) OR*
    *(TEST_DEF.TEST_CLASS = TEST_CLASS_DEF.AXIS_PERIODIC) OR*
    *(TEST_DEF.TEST_CLASS = TEST_CLASS_DEF.THERMAL_AXIS)) AND*
    *(EXISTS(TEMP_MATERIAL_COMP)));*
   *WR3: ((TEMP_MATERIAL_COMP) AND*
    *((EXISTS(TEMP_MATERIAL_SENSOR) AND*
    *EXISTS(TEMP_MATERIAL_COEFFICIENT))));*
   *WR4: ((MEAS_METHOD = MEAS_METHOD_DEF.DIFFERENTIAL) AND*
    *(EXISTS(DIFFERENTIAL_MEAS_DIR)));*
*END_ENTITY;*
*ENTITY SETUP_LINE_STATIC_DEF*
 *SUBTYPE OF (SETUP_LINE_DEF);*
  *SETUP_STATIC: SETUP_STATIC_DEF;*
*END_ENTITY;*
*ENTITY SETUP_LINE_DYNAMIC_DEF*
 *SUBTYPE OF (SETUP_LINE_DEF);*
  *SETUP_DYNAMIC: SETUP_DYNAMIC_DEF;*
*END_ENTITY;*
*ENTITY ALIGNMENT_DEF;*
 *AXIS_SECOND: AXIS_DEF;*
 *TARGETS_SECOND: OPTIONAL LIST[1:?] OF REAL;*
 *TARGET_START_SECOND: OPTIONAL REAL;*
 *TARGET_END_SECOND: OPTIONAL REAL;*
 *MACHINING_TIME: OPTIONAL REAL;*
 *SOAK_OUT_TIME: OPTIONAL REAL;*
  *WHERE*
  *WR1: (((SETUP_LINE_DEF.MEAS_MODE = MEAS_MODE_DEF.STATIC) AND*
    *(SETUP_LINE_DEF.MEAS_METHOD = MEAS_METHOD_DEF.SQUARE)) AND*
    *(EXISTS (TARGETS_SECOND)));*
   *WR2: (((SETUP_LINE_DEF.MEAS_MODE = MEAS_MODE_DEF.DYNAMIC) AND*
    *(SETUP_LINE_DEF.MEAS_METHOD = MEAS_METHOD_DEF.SQUARE)) AND*
    *(EXISTS (TARGET_START_SECOND) AND EXISTS (TARGET_END_SECOND)));*
   *WR3: (((SETUP_LINE_DEF.MEAS_METHOD = MEAS_METHOD_DEF.PAST_CENTER) OR*
    *(SETUP_LINE_DEF.MEAS_METHOD = MEAS_METHOD_DEF.REVERSE_PART)) AND*
    *(EXISTS (MACHINING_TIME) AND EXISTS (SOAK_OUT_TIME)));*
*END_ENTITY;*
*ENTITY RUN_DATA_LINE_DEF*
 *SUBTYPE OF (RUN_DATA_DEF);*
   *APPROACH_DIRECTION: APPROACH_DIRECTION_DEF;*
   *LEG: OPTIONAL LEG_DEF;*
   *LS_OFFSET: OPTIONAL REAL;*
   *LS_CENTER_OFFSET_X: OPTIONAL REAL;*
   *LS_CENTER_OFFSET_Y: OPTIONAL REAL;*
   *LS_SLOPE: OPTIONAL REAL;*
   *TEMP_REFERENCE: OPTIONAL LIST [1:?] OF TEMP_DATA_DEF;*
   *TEMP_MATERIAL: OPTIONAL LIST [1:?] OF TEMP_DATA_DEF;*
   *TEMP_ADDITIONAL: OPTIONAL LIST [1:?] OF TEMP_DATA_DEF;*
   *AIR_HUMIDITY: OPTIONAL LIST [1:?] OF REAL;*
   *AIR_PRESSURE: OPTIONAL LIST [1:?] OF REAL;*
   *ELAPSED_TIME: OPTIONAL REAL;*
   *POINTS: OPTIONAL LIST[1:?] OF REAL;*
    *WHERE*
    *WR1: ((EQUIPMENT_LINE_DEF.FILTER_OFFSET) AND EXISTS(LS_OFFSET));*
    *WR2: ((EQUIPMENT_LINE_DEF.FILTER_LS_CENTER) AND*
     *(EXISTS(LS_CENTER_OFFSET_X) AND EXISTS(LS_CENTER_OFFSET_Y)));*
    *WR3: ((EQUIPMENT_LINE_DEF.FILTER_LS_SLOPE) AND EXISTS(LS_SLOPE));*
    *WR4: (((EQUIPMENT_LINE_DEF.EQUIPMENT_CLASS =*
     *EQUIPMENT_CLASS_LINE_DEF.LASER_INTERFEROMETER) OR*
     *(EQUIPMENT_LINE_DEF.EQUIPMENT_CLASS =*
     *EQUIPMENT_CLASS_LINE_DEF.ND_LASER)) AND*
     *(EXISTS(AIR_HUMIDITY)) AND (EXISTS(AIR_PRESSURE)));*
*END_ENTITY;*
*ENTITY EQUIPMENT_POINT_DEF*
 *SUBTYPE OF (EQUIPMENT_DEF);*
  *EQUIPMENT_CLASS: OPTIONAL EQUIPMENT_CLASS_POINT_DEF;*
  *CUT_OFF: OPTIONAL REAL;*
  *SYNCHRONIZATION: OPTIONAL SYNCHRONIZATION_DEF;*
  *TEMP_REFERENCE_COEFFICIENT: OPTIONAL REAL;*
  *FILTER_LS_CENTER: OPTIONAL BOOLEAN;*
  *TARGET_SHAPE : OPTIONAL TARGET_SHAPE_DEF;*
  *TARGET_DIAMETER : OPTIONAL REAL;*
  *DUAL_SENSOR : OPTIONAL BOOLEAN;*
*END_ENTITY;*
*ENTITY SETUP_POINT_DEF*
 *SUBTYPE OF (SETUP_DEF);*
  *ID: OPTIONAL STRING;*
  *MEAS_MODE: OPTIONAL MEAS_MODE_DEF;*
  *MEASURAND: LIST [1:?] OF MEASURAND_POINT_DEF;*
  *SENSOR_OFFSET: OPTIONAL REAL;*
  *MACHINE_POSITION: MACHINE_POSITION_DEF;*
  *TOOL_VECTOR: TOOL_VECTOR_DEF;*
  *SPINDLE_NUMBER: OPTIONAL INTEGER;*
  *TURRET_NUMBER: OPTIONAL INTEGER;*
  *TEMP_ADDITIONAL_SENSOR: OPTIONAL LIST [1:?] OF TEMP_SENSOR_DEF;*
  *SAMPLE_RATE: REAL;*
  *SAMPLES_AVERAGED: OPTIONAL INTEGER;*
  *SENSITIVE_DIRECTION: OPTIONAL SENSITIVE_DIRECTION_DEF;*
  *NUMBER_OF_REVOLUTIONS: OPTIONAL INTEGER;*
  *AXIS: OPTIONAL AXIS_DEF;*
  *SPINDLE_SPEED: OPTIONAL LIST[1:?] OF REAL;*
  *DURATION: OPTIONAL LIST[1:?] OF REAL;*
  *TOOL_LENGTH_LONG: OPTIONAL REAL;*
  *FEEDRATE: OPTIONAL REAL;*
  *APPROACH_POINT: OPTIONAL MACHINE_POSITION_DEF;*
  *SETUP_STATIC: OPTIONAL SETUP_STATIC_DEF;*
*END_ENTITY;*
*ENTITY RUN_DATA_POINT_DEF*
 *SUBTYPE OF (RUN_DATA_DEF);*
*TEMP*  *_ADDITIONAL: OPTIONAL LIST [1:?] OF TEMP_DATA_DEF;*
  *LS_CENTER_OFFSET_X: OPTIONAL REAL;*
  *LS_CENTER_OFFSET_Y: OPTIONAL REAL;*
  *POINTS: LIST[1:?] OF REAL;*
   *WHERE*
   *WR1: ((EQUIPMENT_POINT_DEF.FILTER_LS_CENTER) AND*
    *(EXISTS(LS_CENTER_OFFSET_X) AND EXISTS (LS_CENTER_OFFSET_Y)));*
*END_ENTITY;*
*ENTITY EQUIPMENT_COMPLIANCE_DEF*
 *SUBTYPE OF (EQUIPMENT_DEF);*
  *EQUIPMENT_CLASS: OPTIONAL EQUIPMENT_CLASS_COMPLIANCE_DEF;*
  *LOAD_MEASUREMENT: OPTIONAL LOAD_MEASUREMENT_DEF;*
  *LOAD_RESOLUTION: OPTIONAL REAL;*
*END_ENTITY;*
*ENTITY SETUP_COMPLIANCE_DEF*
 *SUBTYPE OF (SETUP_DEF);*
  *ID: OPTIONAL STRING;*
  *AXIS: AXIS_DEF;*
  *EXTERNAL_LOAD: BOOLEAN;*
  *AXIS_LOAD: OPTIONAL AXIS_DEF;*
  *MEAS_DIR: OPTIONAL MEAS_DIR_DEF;*
  *RADIUS: OPTIONAL REAL;*
  *MACHINE_POSITION: MACHINE_POSITION_DEF;*
  *TOOL_VECTOR: TOOL_VECTOR_DEF;*
  *SPINDLE_NUMBER: OPTIONAL INTEGER;*
  *TURRET_NUMBER: OPTIONAL INTEGER;*
  *SAMPLES_AVERAGED : OPTIONAL INTEGER;*
*END_ENTITY;*
*ENTITY RUN_DATA_COMPLIANCE_DEF*
 *SUBTYPE OF (RUN_DATA_DEF);*
  *POINTS: LIST[1:?] OF REAL;*
*END_ENTITY;*
*ENTITY AXIS_POSITION_DEF;*
 *AXIS: AXIS_DEF;*
 *POSITION: REAL;*
*END_ENTITY;*
*ENTITY MACHINE_POSITION_DEF;*
 *POSITIONS: LIST [1:?] OF AXIS_POSITION_DEF;*
*END_ENTITY;*
*ENTITY SETUP_DYNAMIC_DEF;*
 *TARGET_START: REAL;*
 *TARGET_END: REAL;*
 *TRIGGER_MODE: OPTIONAL TRIGGER_MODE_DEF;*
 *SAMPLE_RATE: OPTIONAL REAL;*
 *INFEED_MODE: OPTIONAL INFEED_MODE_DEF;*
 *INFEED_DISTANCE: OPTIONAL REAL;*
 *INFEED_RADIUS: OPTIONAL REAL;*
 *INFEED_ANGLE: OPTIONAL REAL;*
  *WHERE*
  *WR1: ((INFEED_MODE = INFEED_MODE_DEF.LINEAR) AND*
   *(EXISTS (INFEED_DISTANCE)));*
  *WR2: ((INFEED_MODE = INFEED_MODE_DEF.CIRCULAR) AND*
   *(EXISTS (INFEED_RADIUS) AND EXISTS (INFEED_ANGLE)));*
*END_ENTITY;*
*ENTITY SETUP_STATIC_DEF;*
 *TARGETS: OPTIONAL LIST [1:?] OF REAL;*
 *REPETITIONS: OPTIONAL INTEGER;*
 *TRIGGER_MODE: OPTIONAL TRIGGER_MODE_DEF;*
 *TRIGGER_DWELL: OPTIONAL REAL;*
 *TRIGGER_WIDTH: OPTIONAL REAL;*
 *TRIGGER_STABILITY: OPTIONAL REAL;*
*END_ENTITY;*
*ENTITY TEMP_SENSOR_DEF;*
 *NAME: OPTIONAL STRING;*
 *LOCATION: STRING;*
 *CHANNEL: OPTIONAL STRING;*
 *SERIAL_NUMBER: OPTIONAL STRING;*
*END_ENTITY;*
*ENTITY TEMP_DATA_DEF;*
 *NAME: OPTIONAL STRING;*
 *DATA: LIST [1:?] OF REAL;*
*END_ENTITY;*
*ENTITY TOOL_VECTOR_DEF;*
 *X: OPTIONAL REAL;*
 *Y: OPTIONAL REAL;*
 *Z: REAL;*
*END_ENTITY;*
*END_SCHEMA;—END MACHINE_TOOL_PERFORMANCE_TESTS*
 (*


#### 2.2.2 Type Definitions

The types are formally defined in this subsection and they are presented in alphabetical order.


 *)
*TYPE ALIGNMENT_METHOD_DEF = ENUMERATION OF*
  *(NO_ALIGN, KINEMATIC, QUADRANT, PROBE, GRID_ENCODER_ZERO);*
*END_TYPE;*
*TYPE ALIGNMENT_WHEN_DEF = ENUMERATION OF*
  *(PREVIOUS, FIRST_RUN, EACH_RUN);*
*END_TYPE;*
*TYPE APPROACH_DIRECTION_DEF = ENUMERATION OF*
  *(POSITIVE, NEGATIVE, BIDIRECTIONAL, PILGRIM_POSITIVE*,
  *PILGRIM_NEGATIVE);*
*END_TYPE;*
*TYPE APPROACH_MODE_CIRCULAR_DEF = ENUMERATION OF*
  *(AXIS, TANGENT, RADIAL);*
*END_TYPE;*
*TYPE AXIS_DEF= STRING;*
*END_TYPE;*
*TYPE DATUM_WHEN_DEF = ENUMERATION OF*
 *(PREVIOUS, FIRST_RUN, EACH_RUN);*
*END_TYPE;*
*TYPE DIFFERENTIAL_MEAS_DIR_DEF = ENUMERATION OF*
 *(X, Y, Z);*
*END_TYPE;*
*TYPE DRIVE_STATUS_DEF = ENUMERATION OF*
  *(OFF, HOLD, PROG);*
*END_TYPE;*
*TYPE EQUIPMENT_CLASS_COMPLIANCE_DEF = ENUMERATION OF*
  *(AUTOCOLLIMATOR, CAPACITANCE, INDUCTIVE, LASER_INTERFEROMETER*,
  *LEVELS, LVDT, MECHANICAL, ND_LASER, SCALE, TRIANGULATION);*
*END_TYPE;*
*TYPE EQUIPMENT_CLASS_CIRCULAR_DEF = ENUMERATION OF*
  *(BALL_BAR, DISK, GRID_ENCODER);*
*END_TYPE;*
*TYPE EQUIPMENT_CLASS_LINE_DEF = ENUMERATION OF*
  *(ALIGNMENTLASER, AUTOCOLLIMATOR, DISPLACEMENT*,
  *INDEXING_AUTOCOLLIMATOR, INDEXING_LEVELS*,
  *INDEXING_LASER_INTERFEROMETER, INDEXING_DISPLACEMENT*,
  *LASER_BALL_BAR, LASER_INTERFEROMETER, LEVELS, MANDREL*,
  *ND_LASER, POLYGON_AUTOCOLLIMATOR*,
  *POLYGON_LASER_INTERFEROMETER, POLYGON_ND_LASER*,
  *ROTARY_ENCODER, SCALE, STRAIGHTEDGE, WIRE);*
*END_TYPE;*
*TYPE EQUIPMENT_CLASS_POINT_DEF = ENUMERATION OF*
  *(INDUCTIVE, CAPACITANCE, LASER_INTERFEROMETER, LVDT, MECHANICAL*,
  *SCALE, TRIANGULATION);*
*END_TYPE;*
*TYPE INFEED_MODE_DEF= ENUMERATION OF*
  *(CIRCULAR, LINEAR, NONE);*
*END_TYPE;*
*TYPE INTERPOLATION_DEF = ENUMERATION OF*
  *(CIRCULAR, LINEAR);*
*END_TYPE;*
*TYPE LEG_DEF = ENUMERATION OF*
  *(FIRST, SECOND);*
*END_TYPE;*
*TYPE LOAD_MEASUREMENT_DEF = ENUMERATION OF*
  *(FORCE, MOMENT);*
*END_TYPE;*
*TYPE MEAS_DIR_DEF = ENUMERATION OF*
  *(X, Y, Z, A, B, C);*
*END_TYPE;*
*TYPE MEAS_METHOD_DEF = ENUMERATION OF*
  *(DIFFERENTIAL, DIRECT, REVERSE, SQUARE, PAST_CENTER*,
  *REVERSE_PART, TWO_CIRCLE);*
*END_TYPE;*
*TYPE MEAS_MODE_DEF = ENUMERATION OF*
  *(STATIC, DYNAMIC);*
*END_TYPE;*
*TYPE MEASURAND_LINE_DEF = ENUMERATION OF*
  *(A, B, C, X, Y, Z, RA, RR, RT, DV, DA);*
*END_TYPE;*
*TYPE MEASURAND_POINT_DEF = ENUMERATION OF*
  *(X, Y, Z, XS, YS, ZS, A, B, C, RR, RA, RT);*
*END_TYPE;*
*TYPE POINT_MODE_CIRCULAR_DEF= ENUMERATION OF*
  *(R, AR, XY);*
*END_TYPE;*
*TYPE SENSITIVE_DIRECTION_DEF = ENUMERATION OF*
  *(FIXED_DIR, ROTATING);*
*END_TYPE;*
*TYPE STANDARD_STATUS_DEF = ENUMERATION OF*
  *(DRAFT, FINAL);*
*END_TYPE;*
*TYPE SYNCHRONIZATION_DEF = ENUMERATION OF*
  *(ECCENTRICITY, MARKER, MACHINE, NONE);*
*END_TYPE;*
*TYPE TARGET_SHAPE_DEF = ENUMERATION OF*
  *(SPHERE, CYLINDER);*
*END_TYPE;*
*TYPE TEST_CLASS_DEF = ENUMERATION OF*
  *(AXIS_ACCELERATION, AXIS_ANGULAR, AXIS_PERIODIC, AXIS_POSITION*,
  *AXIS_REPEAT, AXIS_REVERSAL, AXIS_STRAIGHT, AXIS_VELOCITY*,
  *CIRCULAR, COMPLIANCE, DIAGONAL_ACCELERATION, DIAGONAL_ANGULAR*,
  *DIAGONAL_POSITION, DIAGONAL_STRAIGHT, DIAGONAL_VELOCITY*,
  *PARALLELISM, SPINDLE, SQUARENESS, STRUCTURAL, SUBSYSTEM_GAGE*,
  *SUBSYSTEM_PALLET, SUBSYSTEM_TOOL, SUBSYSTEM_TURRET*,
  *THERMAL_AXIS, THERMAL_COMPOSITE, THERMAL_ETVE*,
  *THERMAL_SPRINDLE);*
*END_TYPE;*
*TYPE TEXT = STRING;*
*END_TYPE;*
*TYPE TRIGGER_MODE_DEF = ENUMERATION OF*
  *(INFEED, MACHINE, MANUAL, STABILITY, TARGET, TARGET_STABILITY, TIME);*
*END_TYPE;*
*TYPE VOL_COMP_METHOD_DEF = ENUMERATION OF*
  *(MANUAL, SENSOR, TRACKER);*
*END_TYPE;*
*TYPE WHY_DEF = ENUMERATION OF*
  *(ACCEPTANCE, COLLISION, MAINTENANCE, MOVE, PERIODIC);*
*END_TYPE;*
 (*


## 3. Implementation Samples

The example shown in this section is for a dynamic, circular test in the *XY*-plane of a milling machine. The measurements are performed with a ball bar. The programmed circle consists of line segments (an option mentioned in Appendix E8.2 of the ASME B5.57 standard on turning centers [5]). A calibrator is previously used to determine the absolute length of the ball bar.

Three implementation samples on the EXPRESS model are presented for the same data. Section 3.1 demonstrates the implementation using the ISO 10303-21 Exchange Structure [9]. Section 3.2 demonstrates an XML (the Extensible Markup Language) [10] implementation. Section 3.3 demonstrates the implementation using a relational database. All samples have been generated manually.

### 3.1 ISO 10303 Part 21 Exchange Structure

ISO 10303-21 specifies an exchange structure of product data for which the conceptual model is specified in the EXPRESS language. The file format is suitable for transfer among computer systems. The exchange structure is designed to facilitate parsing by software.

The following is a sample of an exchange structure based on the ISO 10303-21, Clear Text Encoding Of the Exchange Structure [9]. Each *Part 21* file format may be considered a continuous stream. This exchange structure consists of two sections: the header section and the data section. The header section contains information that is applicable to the entire exchange file. The data section contains instances of entities that correspond to the EXPRESS schema governing the exchange structure as specified in the header section. An entity instance name is identified by a *number sign* (*#*), followed by a unique entity name, which is an unsigned integer of 1 or more digits. When a value is not provided for an optional attribute, the attribute value is encoded as the *dollar sign* ($). Both forward and backward references are permitted. A comment is encoded as a *solidus asterisk* (/*) followed by any number of characters, and terminated by an *asterisk solidus* (*/).


/*
*The exchange file is generated based on the ISO 10303-21: 1994(E).*
*The file has been presented in a line-oriented or record-oriented manner in order to aid readability.*
*Unnecessary spaces have been added to aid readability.*
*Note that an ordinary Part 21 file is not aligned in this manner, but is a continuous stream of characters.*
*/
/*
*The following gives the short names for the schema of MACHINE_TOOL_PERFORMANCE_TESTS.*
*Entity name*                   *Short name*
*BALL_BAR_DEF*                *BALL_BAR*
*COMPONENT_DEF*              *COMPONENT*
*CONDITIONS_DEF*             *CONDITIONS*
*DATE_DEF*                  *DATE*
*MACHINE_DEF*               *MACHINE*
*PLANE_DEF*                 *PLANE*
*MACHINE_POSITION_DEF*         *MACHINE_POSITION*
*RESULT_DEF*                 *RESULT*
*RUN_DATA_CIRCULAR_DEF*       *RUN_DATA_CIRCULAR*
*SETUP_CIRCULAR_DYNAMIC_DEF*   *SETUP_CIRCULAR_DYNAMIC*
*SETUP_DYNAMIC_DEF*          *SETUP_DYNAMIC*
*SOFTWARE_DEF*              *SOFTWARE*
*STANDARD_DEF*              *STANDARD*
*TEMP_SENSOR_DEF*            *TEMP_SENSOR*
*TEST_DEF*                  *TEST*
*TIME_DEF*                  *TIME*
*TOOL_VECTOR_DEF*            *TOOL_VECTOR*
*/
*ISO-10303-21;*
*HEADER;*
*FILE_DESCRIPTION ((‘THIS FILE CONTAINS A SAMPLE CIRCULAR TEST’), ‘2’);*
*FILE_NAME (‘EXAMPLE PART 21 FILE #1’, ‘2000-07-17T17:30:00’*,
*(‘TINA LEE’,‘NIST’,‘MS8260’,‘Gaithersburg, MD 20899-8260’)*,
*(‘NIST/MEL/MSG’), ‘PREPROCESSOR_VERSION NONE’*,
*‘ORIGINATING SYSTEM RELEASE 1.0’, ‘APPROVED BY TINA LEE’);*
*FILE_SCHEMA ((‘MACHINE_TOOL_PERFORMANCE_TESTS’));*
*ENDSEC;*
*DATA;*
*#1=DATE(1999,6,22);*
*#2=TIME(10, 6, 0);*
*#3=MACHINE(‘2434’,‘XYZ’,‘ABC’,‘123’,‘SHOPS’);*
*#4=CONDITIONS($,.T.,$,$,$,22.5,$,$);*
*#5=STANDARD(‘ASME’,‘B5.57’,$,1997,$,$,$,$);*
*#6=COMPONENT($,‘BALL_BAR’,‘XYZ’,‘ABC1’,‘123’,$,$,$,$);*
*#7=COMPONENT($,‘CALIBRATOR’,‘XYZ’,‘ABC2’,‘456’,$,$,$,$);*
*#8=SOFTWARE($,‘XYZ’,‘ABC3’,‘3.0’);*
*#9=BALL_BAR(‘BALL BAR BOX 123’,(#6, #7), #8,0.1,$, .BALL_BAR.,.T.,.F.,.F.,.F.,$,0.5,.T.);*
*#10=PLANE((.X.,$),(.Y.,$));*
*#11=MACHINE_POSITION(((‘X’,400.0),(‘Y’,350.0),(‘Z’,100.0)));*
*#12=TOOL_VECTOR(0,0,-100.0);*
*#13=SETUP_DYNAMIC(0, 360.0, $,,125.0, $,$,$,$);*
*#14=SETUP_CIRCULAR_DYNAMIC(‘2434’,.DYNMAIC., #10,$, #11, #12,1,$,150.0,150.0*,
 *0,1500.0,180.0,.T.,(($,’TABLE’,$,$)),11.5,$,.R.,.LINEAR.,$,.KINEMATIC.*,
 *$,$,$,#13,$,0.5);*
*#15=RUN_DATA_CIRCULAR(.POSITIVE.,5.0,22.0,122.0,$,(($,(22.4)))*,
 *(1.5,0.5,0.6,0.2,0.4,....));*
*#16=RUN_DATA_CIRCULAR(.NEGATIVE.,8.0,24.0,112.0,$,(($,(22.4)))*,
 *(0.5,0.5,0.6,0.2,0.4,...));*
*#17=RESULT(#5,$,((‘LS_RADIUS_ERROR’,122.0,.POSITIVE.)*,
 *(‘CIRCULARITY’,11.0,.POSITIVE.)));*
*#18=RESULT(#5,$,((‘LS_RADIUS_ERROR’,112.0,.NEGATIVE.)*,
 *(‘CIRCULARITY’,14.0,.NEGATIVE.)));*
*#19=RESULT(#5,$,((‘LS_RADIUS_ERROR’,117.0,.BIDIRECTIONAL.)*,
 *(‘CIRCULARITY’,22.0,.BIDIRECTIONAL.)));*
*#20=STANDARD(‘ISO’,’230-4’,$,1996,$,$,$,$);*
*#21=RESULT(#20,$,((‘LS_RADIUS_ERROR’,117.0,.BIDIRECTIONAL.)*,
 *(‘CIRCULARITY’,22.0,.BIDIRECTIONAL.),(‘HYSTERESIS’,12.0,.BIDIRECTIONAL.)));*
*#22=TEST(‘BB0699A.RTB’,.CIRCULAR.,#1,#2,.PERIODIC.,#3,#4,‘JOHN DOE’,#5*,
 *#9,#14,(#15,#16),(#17,#18,#19,#21),‘THIS IS AN EXAMPLE’);*
*ENDSEC;*
*END-ISO-10303-21;*


### 3.2 XML Document

XML, an extensible markup language, is a universal format for structured documents and data on the Web [10]. The language helps make information exchange among a globally distributed computing environment possible. XML allows precise encoding of structured information. The XML data file (below) contains both data and an indication of the meaning of the data. XML is for the digital representation of documents.

The following is an XML document sample. This XML document is well-formed, which means that the tags are properly constructed. This XML document, however, does not contain a document type definition^2^(DTD). Our intention for this subsection is to demonstrate the XML implementation of the EXPRESS model, the development of the DTD will be the topic for another report. An XML document is composed of a series of characters. It has two main parts: a prolog and a document instance. The prolog is optional, and describes the XML version, DTD, and other characteristics of the document. The document instance follows the prolog and contains the actual document data organized as a hierarchy of elements. An XML source is made up of XML elements, each of which consists of a start-tag, the element content, and an end-tag. An XML start-tag consists of the *less-than* symbol (<), the name of the element, and a *greater-than* symbol (>). Start-tags can also include attributes. An XML end-tag consists of the string “</”, the same element name as in the start-tag, and a *greater-than* symbol (>).


*<?xml version =”1.0”?>*
*<TEST>*
 *<ID> BB0699A.RTB</ID>*
 *<TEST_CLASS>CIRCULAR</TEST_CLASS>*
 *<DATE><YYYY>1999</YYYY><MM>06</MM><DD>22</DD></DATE>*
 *<TIME><HH>10</HH><MM>06</MM><SS>00</SS></TIME>*
 *<WHY>PERIODIC</WHY>*
 *<MACHINE>*
   *<ID>2434</ID>*
   *<MANUFACTURER>XYZ</MANUFACTURER>*
   *<MACHINE_MODEL>ABC</MACHINE_MODEL>*
   *<SERIAL_NUMBER>123</SERIAL_NUMBER>*
   *<LOCATION>SHOPS</LOCATION>*
 *</MACHINE>*
 *<CONDITIONS>*
   *<COMPENSATION>YES</COMPENSATION>*
   *<TEMP_ENVIRONMENT>22.5</TEMP_ENVIRONMENT>*
 *</CONDITIONS>*
 *<OPERATOR> JOHN DOE</OPERATOR>*
 *<STANDARD>*
  *<ORGANIZATION>ASME</ORGANIZATION>*
  *<STANDARD_NUMBER>B5.57</STANDARD_NUMBER>*
  *<YEAR>1997</YEAR>*
 *</STANDARD>*
 *<EQUIPMENT>*
  *<ID>BALL BAR BOX 123</ID>*
  *<COMPONENT>*
   *<DESCRIPTION>BALLBAR</DESCRIPTION>*
   *<MANUFACTURER>XYZ</MANUFACTURER>*
   *<COMPONENT_MODEL>ABC1</COMPONENT_MODEL>*
   *<SERIAL_NUMBER>123</SERIAL_NUMBER>*
 *</COMPONENT>*
 *<COMPONENT>*
  *<DESCRIPTION>CALIBRATOR</DESCRIPTION>*
  *<MANUFACTURER>XYZ</MANUFACTURER>*
  *<COMPONENT_MODEL>ABC2</COMPONENT_MODEL>*
 *<SERIAL_NUMBER>456</SERIAL_NUMBER>*
 *</COMPONENT>*
 *<SOFTWARE>*
  *<MANUFACTURER>XYZ</MANUFACTURER>*
  *<NAME>ABC3</NAME>*
  *<VERSION_NUMBER>3.0</VERSION_NUMBER>*
 *</SOFTWARE>*
 *<RESOLUTION>0.1</RESOLUTION>*
 *<EQUIPMENT_CLASS>BALL_BAR</EQUIPMENT_CLASS>*
 *<ABSOLUTE>YES</ABSOLUTE>*
 *<FILTER_LS_CENTER>NO</FILTER_LS_CENTER>*
 *<FILTER_LS_RADIUS>NO</FILTER_LS_RADIUS>*
 *<TEMP_REFERENCE_COMP>NO</TEMP_REFERENCE_COMP>*
 *<TEMP_REFERENCE_COEFFICIENT>0.5</TEMP_REFERENCE_COEFFICIENT>*
 *<CALIBRATOR>YES</CALIBRATOR>*
 *</EQUIPMENT>*
 *<SETUP>*
  *<ID>2434</ID>*
  *<MEAS_MODE>DYNAMIC</MEAS_MODE>*
  *<PLANE><X>X</X><Y>Y</Y></PLANE>*
  *<CENTER>*
  *<AXIS_POSITION>*
   *<AXIS>X</AXIS><POSITION>400</POSITION>*
  *</AXIS_POSITION>*
  *<AXIS_POSITION>*
   *<AXIS>Y</AXIS><POSITION>350</POSITION>*
  *</AXIS_POSITION>*
  *<AXIS_POSITION>*
   *<AXIS>Z</AXIS><POSITION>100</POSITION>*
  *</AXIS_POSITION>*
  *</CENTER>*
  *<TOOL_VECTOR><X>0</X><Y>0</Y><Z>-100</Z></TOOL_VECTOR>*
  *<SPINDLE_NUMBER>1</SPINDLE_NUMBER>*
  *<RADIUS_MACHINE>150</RADIUS_MACHINE>*
  *<RADIUS_REFERENCE>150</RADIUS_REFERENCE>*
  *<INCLINATION>0</INCLINATION>*
  *<FEEDRATE>1500</FEED_RATE>*
  *<OVERSHOOT>180</OVER_SHOOT>*
  *<TEMP_MATERIAL_COMP>YES</TEMP_MATERIAL_COMP>*
  *<TEMP_MATERIAL_SENSOR>*
   *<LOCATION>TABLE</LOCATION></TEMP_MATERIAL_SENSOR>*
  *<TEMP_MATERIAL_COEFFICIENT>11.5</TEMP_MATERIAL_COEFFICIENT>*
  *<POINT_MODE>R</POINT_MODE>*
  *<INTERPOLATION>LINEAR</INTERPOLATION>*
  *<ALIGNMENT_METHOD>KINEMATIC</ALIGNMENT_METHOD>*
  *<SETUP_DYNAMIC>*
    *<TARGET_START>0</TARGET_START><TARGET_END>360</TARGET_END>*
    *<SAMPLE_RATE>125</SAMPLE_RATE></SETUP_DYNAMIC>*
  *<NC_CIRCULARITY>0.5</NC_CIRCULARITY>*
 *</SETUP>*
 *<RUN_DATA>*
  *<APPROACH_DIRECTION>POSITIVE</APPROACH_DIRECTION>*
  *<LS_CENTER_OFFSET_X>5</LS_CENTER_OFFSET_X>*
  *<LS_CENTER_OFFSET_Y>22</LS_CENTER_OFFEST_Y>*
  *<LS_RADIUS_ERROR>122</LS_RADIUS_ERROR>*
  *<TEMP_MATERIAL><DATA>22.4</DATA></TEMP_MATERIAL>*
  *<POINTS>1.5 0.5 0.6 0.2 0.4 ….. </POINTS>*
 *</RUN_DATA>*
 *<RUN_DATA>*
  *<APPROACH_DIRECTION>NEGATIVE</APPROACH_DIRECTION>*
  *<LS_CENTER_OFFSET_X>8</LS_CENTER_OFFSET_X>>*
  *<LS_CENTER_OFFSET_Y>24</LS_CENTER_OFFSET_Y>*
  *<LS_RADIUS_ERROR>112</LS_RADIUS_ERROR>*
  *<TEMP_MATERIAL><DATA>22.4</DATA></TEMP_MATERIAL>*
  *<POINTS>0.5 0.5 0.6 0.2 0.4 ….. </POINTS>*
 *</RUN_DATA>*
 *<RESULT>*
  *<STANDARD>*
   *<ORGANIZATION>ASME</ORGANIZATION>*
   *<STANDARD_NUMBER>B5.57</STANDARD_NUMBER><YEAR>1997</YEAR>*
  *</STANDARD>*
  *<PARAMETER>*
   *<NAME>LS_RADIUS_ERROR</NAME><VAL>122</VAL>*
   *<APPROACH_DIRECTION>POSITIVE</APPROACH_DIRECTION>*
  *</PARAMETER>*
  *<PARAMETER>*
   *<NAME>CIRCULARITY</NAME><VAL>11</VAL>*
   *<APPROACH_DIRECTION>POSITIVE</APPROACH_DIRECTION>*
  *</PARAMETER>*
 *</RESULT>*
 *<RESULT>*
  *<STANDARD>*
   *<ORGANIZATION>ASME</ORGANIZATION>*
   *<STANDARD_NUMBER>B5.57</STANDARD_NUMBER><YEAR>1997</YEAR>*
  *</STANDARD>*
  *<PARAMETER>*
   *<NAME>LS_RADIUS_ERROR</NAME><VAL>112</VAL>*
   *<APPROACH_DIRECTION>NEGATIVE</APPROACH_DIRECTION>*
  *</PARAMETER>*
  *<PARAMETER>*
   *<NAME>CIRCULARITY</NAME><VAL>14</VAL>*
   *<APPROACH_DIRECTION>NEGATIVE</APPROACH_DIRECTION>*
  *</PARAMETER>*
 *</RESULT>*
 *<RESULT>*
  *<STANDARD>*
   *<ORGANIZATION>ASME</ORGANIZATION>*
   *<STANDARD_NUMBER>B5.57</STANDARD_NUMBER><YEAR>1997</YEAR>*
  *</STANDARD>*
  *<PARAMETER>*
   *<NAME>LS_RADIUS_ERROR</NAME><VAL>117</VAL>*
   *<APPROACH_DIRECTION>BIDIRECTIONAL</APPROACH_DIRECTION>*
  *</PARAMETER>*
  *<PARAMETER>*
   *<NAME>CIRCULARITY</NAME><VAL>22</VAL>*
   *<APPROACH_DIRECTION>BIDIRECTIONAL</APPROACH_DIRECTION>*
  *</PARAMETER>*
 *</RESULT>*
 *<RESULT>*
  *<STANDARD>*
   *<ORGANIZATION>ISO</ORGANIZATION>*
   *<STANDARD_NUMBER>230-4</STANDARD_NUMBER><YEAR>1996</YEAR>*
  *</STANDARD>*
  *<PARAMETER>*
   *<NAME>LS_RADIUS_ERROR</NAME><VAL>117</VAL>*
   *<APPROACH_DIRECTION>BIDIRECTIONAL</APPROACH_DIRECTION>*
  *</PARAMETER>*
  *<PARAMETER>*
   *<NAME>CIRCULARITY</NAME><VAL>22</VAL>*
   *<APPROACH_DIRECTION>BIDIRECTIONAL</APPROACH_DIRECTION>*
  *</PARAMETER>*
  *<PARAMETER>*
   *<NAME>HYSTERESIS</NAME><VAL>12</VAL>*
   *<APPROACH_DIRECTION>BIDIRECTIONAL</APPROACH_DIRECTION>*
  *</PARAMETER>*
 *</RESULT>*
 *<COMMENT>THIS IS AN EXAMPLE</COMMENT>*
*</TEST>*


### 3.3 Relational Tables

Database technology has evolved rapidly. The evolution has moved from simple files to the use of network and hierarchical database management systems, and to today’s relational systems and object-oriented systems. Evolving technology has made data sharing a realistic alternative. Moreover, today’s generation of powerful, inexpensive workstation computers enables users to design software that maintains and distributes data quickly and inexpensively. Relational database management systems are generally desirable for data transfer for the manufacturing community.

All information in a relational database is represented explicitly as values in tables. The Structured Query Language (SQL) [11] was developed to service a relational database. SQL was originally made an ANSI (the American National Standards Institute) standard in 1986, was revised and extended in 1989, and accepted by the ISO in 1992. SQL is a set of commands that are used to create and maintain database tables, manipulate and retrieve data from the relational databases.

The following is a sample of relational tables for the EQUIPMENT entity. These tables have been manually mapped from the respective portion of the EXPRESS information model. Our intention for this subsection is to demonstrate the relational database implementation of the EXPRESS model, the development of the SQL statements that map the complete MACHINE_TOOL_PERFORMANCE_TESTS schema will be the topic for another report. In the SQL statements below, the OID_MAPPING table contains identity information for every EXPRESS object in the database and “OID” stands for Object Identifier.

#### 3.3.1 SQL Statements

**Table:** 

*CREATE TABLE OID_MAPPING (*	
*OID_KEY*	*INTEGER NOT NULL PRIMARY KEY*,
*ENTITY_TYPE*	*VARCHAR(80)*
*);*	
*CREATE TABLE COMPONENT (*	
*COMPONENT_ID*	*INTEGER NOT NULL REFERENCE OID_MAPPING(OID_KEY)*,
*ID*	*VARCHAR(100) NULL*,
*DESCRIPTION*	*VARCHAR(100) NULL*,
*MANUFACTURER*	*VARCHAR(100)*,
*COMPONENT_MODEL*	*VARCHAR(100)*,
*SERIAL_NUMBER*	*VARCHAR(100)*,
*CALIBRATION_DATE*	*VARCHAR(100) NULL*,
*CALIBRATION_EXP_DATE*	*VARCHAR(100) NULL*,
*CERTIFICATE_NUMBER*	*VARCHAR(100) NULL*,
*CALIBRATION_ORGANIZATION*	*VARCHAR(100) NULL*
*);*	
*CREATE TABLE LIST_OF_COMPONENT (*	
*LIST_OF_COMPONENT_ID*	*INTEGER NOT NULL REFERENCE OID_MAPPING(OID_KEY)*,
*COMPONENT_ID*	*INTEGER*,
*COMPONENT_INDEX*	*INTEGER*
*);*	
*CREATE TABLE SOFTWARE (*	
*SOFTWARE_ID*	*INTEGER NOT NULL REFERENCE OID_MAPPING(OID_KEY)*,
*ID*	*VARCHAR(100) NULL*,
*MANUFACTURER*	*VARCHAR(100)*,
*NAME*	*VARCHAR(100)*,
*VERSION_NUMBER*	*VARCHAR(100)*
*);*	
*CREATE TABLE EQUIPMENT (*	
*EQUIPMENT_ID*	*INTEGER NOT NULL REFERENCE OID_MAPPING(OID_KEY)*,
*ID*	*VARCHAR(100) NULL*,
*COMPONENTS_ID*	*INTEGER NULL*,
*SOFTWARE_ID*	*INTEGER NULL*,
*RESOLUTION*	*DOUBLE PRECISION NULL*,
*SAMPLE_RATE_RAW*	*DOUBLE PRECISION NULL*
*);*	
*CREATE TABLE TEMP_SENSOR (*	
*TEMP_SENSOR_ID*	*INTEGER NOT NULL REFERENCE OID_MAPPING(OID_KEY)*,
*NAME*	*VARCHAR(100) NULL*,
*LOCATION*	*VARCHAR(100)*,
*CHANNEL*	*VARCHAR(100) NULL*,
*SERIAL_NUMBER*	*VARCHAR(100) NULL*
*);*	
*CREATE TABLE LIST_OF_TEMP_SENSOR (*	
*LIST_OF_TEMP_SENSOR_ID*	*INTEGER NOT NULL REFERENCE OID_MAPPING(OID_KEY)*,
*TEMP_SENSOR_ID*	*INTEGER*,
*TEMP_SENSOR_INDEX*	*INTEGER*
*);*	
*CREATE TABLE EQUIPMENT_CIRCULAR (*	
*EQUIPMENT_CIRCULAR_ID*	*INTEGER NOT NULL REFERENCE OID_MAPPING(OID_KEY)*,
*EQUIPMENT_CLASS*	*VARCHAR(100)*,
*ABSOLUTE*	*INTEGER*,
*FILTER_LS_CENTER*	*INTEGER*,
*FILTER_LS_RADIUS*	*INTEGER*,
*TEMP_REFERENCE_COMP*	*INTEGER*,
*TEMP_REFERENCE_SENSORS_ID*	*INTEGER NULL*,
*TEMP_REFERENCE_COEFF*	*DOUBLE PRECISION*
*);*	
*CREATE TABLE BALL_BAR (*	
*BALL_BAR_ID*	*INTEGER NOT NULL REFERENCE OID_MAPPING(OID_KEY)*,
*CALIBRATOR*	*INTEGER NULL*
*);*	

#### 3.3.2 Sample Data

## 4. Use of the Information Model

The information model, MACHINE_TOOL_PERFORMANCE_TESTS, presented in Sec. 2 specifies the information necessary to represent the properties and results of machine-tool-performance tests. The model has been successfully parsed using *fedex*, one of the applications in the NIST EXPRESS Toolkit [12]. The NIST EXPRESS Toolkit is a software library for building software tools for manipulating EXPRESS information models, and *fedex* is the tool that reports syntactic and semantic errors in EXPRESS schemas.

The MACHINE_TOOL_PERFORMANCE_TESTS information model is independent of any implementation method. Several commercial and non-commercial software tools exist to support the implementation of EXPRESS information models. A document describing software tools and services for EXPRESS was published by P. R. Wilson [13] and is available from the ISO TC184/SC4 homepage [14]. NIST has released a STEP Toolset for manipulating STEP data; the Toolset is in the public domain and is also available on the ISO TC184/SC4 homepage [14]. The implementors can take advantage of these software tools to generate various types of data structures from the information model in order to benefit the exchange of machine-tool-performance data.

## 5. Conclusion

This report describes the approach being taken by NIST in developing a neutral format for exchanging machine-tool-performance data. An information model of machine-tool-performance tests in EXPRESS has been developed. The implementations of the information model using the STEP exchange structure, XML, and SQL have been demonstrated. The information model will continue to evolve based on experience and feedback from others involved in this effort. Our objective is to promote the information model to an official standard. Broader participation in this effort will help the standardization work proceed more quickly and will also enhance the system performance and user satisfaction.

## Figures and Tables

**Table 1:** Table of OID_MAPPING

*OID-KEY*	*ENTITY-TYPE*
*6*	*“COMPONENT”*
*7*	*“COMPONENT”*
*8*	*“SOFTWARE”*
*9*	*“BALL_BAR”*
*100*	*“LIST_OF_COMPONENT”*

**Table 2:** Table of EQUIPMENT

EQUIPMENT-ID	ID	COMPONENT	SOFTWARE	RESOLUTION	SAMPLE-RATE-RAW
*9*	*“BALL BAR BOX 123”*	*100*	*8*	*0.1*	*–*

**Table 3:** Table of EQUIPMENT_CIRCULAR

EQUIPMENT-CIRCULAR-ID	EQUIPMENT-CLASS	ABSOLUTE	FILTER-LS-CENTER	FILTER-LS-RADIUS
*9*	*“BALL_BAR”*	*1*	*0*	*0*

TEMP-REFERENCE-COMP	TEMP-REFERENCE-SENSOR	TEMP-REFERENCE-COEFFICIENT
*0*	*–*	*0.5*

**Table 4:** Table of COMPONENT

COMPONENT-ID	ID	DESCRIPTION	MANUFACTURER	COMPONENT-MODEL	SERIAL-NUMBER
*6*	*–*	*“BALL_BAR”*	*“XYZ”*	*“ABC1”*	*“123”*
*7*	*–*	*“CALIBRATOR”*	*“XYZ”*	*“ABC2”*	*“456”*

CALIBRATION-DATE	CALIBRATION-EXP-DATE	CERTIFICATE-NUMBER	CALIBRATION-ORGANIZATION
–	–	–	–
–	–	–	–

**Table 5:** Table of LIST_OF_COMPONENT

LIST-OF-COMPONENT-ID	COMPONENT-ID	COMPONENT-INDEX
*100*	*6*	*1*
*100*	*7*	*2*

**Table 6:** Table of SOFTWARE

SOFTWARE-ID	ID	MANUFACTURER	NAME	VERSION-NUMBER
*8*	*–*	*“XYZ”*	*“ABC3”*	*“3.0”*

**Table 7:** Table of BALL_BAR

BALL-BAR-ID	CALIBRATOR
*9*	*1*

## Footnotes

1 EXPRESS-G is represented by graphic symbols forming a diagram. The definitions of data types and schemas within a diagram are denoted by boxes which enclose the name of the item being defined. The relationships between the items are denoted by the lines joining the boxes. Differing line styles provide information on the kind of definition or relationships. For example, a relationship for an optional attribute of an entity data type is presented as a dashed line. An inheritance relationship (i.e., a subtype and supertype relationship) is presented as a thick line.

2 A document type definition (DTD) is the set of rules for using XML to represent documents of a particular type. DTD is a series of definitions for element types, attributes, entities, and notations. DTD is optional for an XML document. Documents that do not have a DTD are not really invalid, but they are not valid either, because they cannot be validated against a DTD.

## 6. Appendix A. List of EXPRESS Key Words

The following is the list of EXPRESS key words that are used in the information model in this report. Brief definitions of these keywords are summarized here for readers’ convenience. For further information, refer to “The EXPRESS Language Reference Manual” [1].

**AND**—The reserve word AND is an AND operator. The AND operator requires two logical operands and evaluates to a logical value.
**BOOLEAN**—A BOOLEAN data type represents a TRUE or FALSE value.
**END_ENTITY**—The key word END_ENTITY is used to terminate an entity declaration.
**END_SCHEMA**—The key word END_SCHEMA is used to terminate a schema declaration.
**END_TYPE**—The key word END_TYPE is used to terminate a type declaration.
**ENTITY**—The key word ENTITY is used to specify an entity type. An entity type characterizes a collection of real-world physical or conceptual objects that have common properties. Any entity declared in a schema can be used as the data type of an attribute, local variable, or parameter. Using an entity as an attribute’s data type establishes a relationship between the two entities.
**ENUMERATION**—The key word ENUMERATION is used to specify an enumeration data type. An enumeration data type is an ordered set of values represented by names. Each enumeration item belongs only to the data type that defines it and must be unique within that type definition.
**FALSE**—The reserve word FALSE is a LOGICAL constant representing the logical notion of falsehood. It is compatible with the BOOLEAN and LOGICAL data types.
**INTEGER**—The key word INTEGER is used to specify an integer data type. An integer data type represents a value of an integer number, the magnitude of which is unconstrained.
**LIST**—The key word LIST is used to specify a list data type. A list data type represents an ordered collections of like elements. The number of elements that can be held in a list can optionally be specified. If the size is not specified, the list can hold any number of elements. Duplicate elements are allowed in a list.
**OF**—The key word OF is used together with other key words such as BAG, LIST, SET, ENUMERATION, SUBTYPE, SUPERTYPE, etc.
**OPTIONAL**—The key word OPTIONAL is used to indicate that the attribute need not have a value in order for an instance of that entity to be valid. In a given entity instance, an attribute marked as optional may have no actual value, in which case the value is said to be null. The null value function (NVL), which returns either the input value or an alternate value in the case where the input has a null value, may be used when a null value is unacceptable.
**OR**—The reserve word OR is an OR operator. The OR operator requires two logical operands and evaluates to a logical value.
**REAL**—The key word REAL is used to specify a real data type. A real data type represents rational, irrational, and scientific real numbers. Rational and irrational numbers have infinite resolution and are exact. Scientific numbers represent values that are known only to a specified precision.
**SCHEMA**—The key word SCHEMA is used to specify a schema type. A schema declaration creates a new scope in which the following objects may be declared: constant, entity, function, procedure, rule, and type.
**STRING**—The key word STRING is used to specify a string data type. A string data type represents a sequence of zero or more characters.
**TRUE**—The reserve word TRUE is a LOGICAL constant representing the logical notion of truth. It is compatible with the BOOLEAN and LOGICAL data types.
**TYPE**—The key word TYPE is used to specify a defined data type. A defined data type is a user extension to the set of standard data types. A defined data type can be used as any other data type by referencing the name given to it.
**UNIQUE**—The key word UNIQUE is used to specify a uniqueness rule. A uniqueness rule specifies either a single attribute name or a list of two or more attribute names. A rule that specifies a single attribute name is a “simple uniqueness constraint’’, requiring that any value of that attribute is associated with only one instance of that entity type. A rule that specifies two or more attribute names is a “joint uniqueness constraint,” requiring that any set of values, one from each of the named attributes, is associated with only one instance of that entity type.
**WHERE**—The key word WHERE is used to specify domain rules. Domain rules constrain the values of individual attributes or combinations of attributes for every entity instance.
